# Functional Imaging of Changes in Lung Function Before and After Radiation Therapy of Lung Cancer

**DOI:** 10.1016/j.adro.2025.101810

**Published:** 2025-06-20

**Authors:** Jonathan L. Percy, Marrissa J. McIntosh, Eric Wallat, Keegan R. Staab, Andrew D. Hahn, Katherine J. Carey, Gregory P. Barton, Andrew M. Baschnagel, John E. Bayouth, Rodrigo M. Bello, Scott B. Perlman, Sean B. Fain

**Affiliations:** a Department of Physics, University of Iowa, Iowa City, Iowa; b Department of Radiology, University of Iowa, Iowa City, Iowa; c Department of Medical Physics, University of Wisconsin-Madison, Madison, Wisconsin; d Department of Human Oncology, University of Wisconsin-Madison, Madison, Wisconsin; e Roy J. Carver Department of Biomedical Engineering, University of Iowa, Iowa City, Iowa; f Department of Radiology, University of Wisconsin – Madison, Madison, Wisconsin; g Department of Internal Medicine, UT Southwestern Medical Center, Dallas, Texas; h Department of Radiation Medicine, Oregon Health and Science University, Portland, Oregon

## Introduction

In 2024, it is predicted that the total number of new respiratory and breast cancer cases will both exceed 250,000.^1^ More than 50% of these patients will receive thoracic radiation therapy (RT) as part of their treatment regime.^2^ Radiation-induced lung injury (RILI) is a serious side effect of radiation treatment^3^ and, as a disease process, encompasses an early (within 1-6 months) inflammatory response known as radiation pneumonitis (RP), and, in a subset of patients, progression to permanent tissue remodeling and fibrosis, ie, radiation pulmonary fibrosis.^2^^,^^4^ Concurrent disease processes in lung cancer patients, including chronic obstructive pulmonary disease and interstitial lung disease (ILD),^4^ make RP difficult to diagnose and may predispose patients to RILI.^4^^,^^5^ The severity of RP is currently determined using grading systems, such as the Common Terminology Criteria for Adverse Events (CTCAE)^6^ and the Radiation Therapy Oncology Group (RTOG) grading system,^7^ which are based on subjective criteria, like the severity of patient-reported symptoms or steroid use, or qualitative imaging.^4^^,^^8^ Variation in RP grading makes it difficult to consistently diagnose and monitor the progression of RP,^8^ thus prioritizing the need for more objective measures. Imaging methods offer a sensitive approach to noninvasively monitor lung pathophysiology indicative of RP to provide earlier detection, guide adjustments to RT dosing, and deploy potential therapies to mitigate RILI and improve outcomes.

Four-dimensional (4D) computed tomography (CT)-derived metrics have been used to describe regional ventilation as well as the response to RT with promising results for functional lung avoidance treatment planning.^9^ Fluorine-18-labeled fluorodeoxyglucose (^18^F-FDG) positron emission tomography (PET) has also been used to evaluate treatment response and RP after RT.^10^^,^^11^ Another emerging clinical technique for imaging lung function is hyperpolarized ^129^Xe (HP-Xe) magnetic resonance imaging (MRI). HP-Xe MRI has previously shown utility in characterizing changes in ventilation, gas exchange, and inflammation of the lungs in healthy controls and in several disease populations, including lung cancer,^12^ and has shown increased sensitivity over chest CT to early changes in normal-appearing lung parenchyma.^13^, ^14^, ^15^, ^16^ Moreover, HP-Xe MRI in conjunction with ultrashort time to echo (UTE) MRI can provide complementary functional and structural information to further support MRI-guided RT in lung cancer.^17^ Here, we examine and compare pulmonary MRI-based measurements longitudinally in a lung cancer patient undergoing stereotactic body RT (SBRT) treatment to demonstrate the feasibility of HP-Xe MRI and UTE MRI for monitoring regional ventilation and gas exchange. An ancillary goal is to show correspondence of pulmonary MRI measures to established methods for evaluating ventilation and inflammation, namely 4DCT and ^18^F-FDG PET/MRI.

## Methods and Materials

### Overview of methods

**Figure 1:**
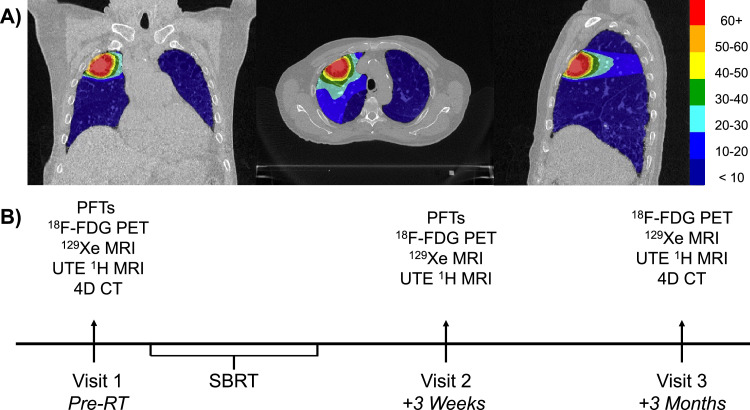
Study timeline and SBRT dose distribution map. Registered dose map overlayed on CT with 10-Gy isodose curves exhibited (A); a total treatment dose of 50 Gy was given in 5 fractions occurring every other day. Visit 1 was conducted prior to SBRT treatment; visit 2 and visit 3 took place 3 weeks and 3 months following the completion of SBRT, respectively (B). PFTs were collected as part of clinical follow-ups which occurred every 3 months (for 12 months) following the conclusion of SBRT. *Abbreviations:* 4DCT = 4-dimensional computed tomography; ^18^F-FDG = fluorodeoxyglucose; PET = positron emission tomography; PFTs = pulmonary function tests; SBRT = stereotactic body radiation therapy; UTE = ultra short time to echo.

A single patient with early-stage non-small cell lung cancer participating in a clinical trial, to investigate the role of functional avoidance in improving outcomes of RP in lung cancer RT (clinicaltrials.gov NCT02843568^18^) was recruited to a more detailed study of functional imaging changes during treatment following approval by the University of Wisconsin Madison institutional review board (IRB#2016-0610). The study was performed in accordance with the Declaration of Helsinki. The study was an exploratory study, and the participant provided written informed consent to participate in this study. The patient was a 70-year-old male diagnosed with stage IA3 (T1c N0 M0) squamous cell carcinoma of the right upper lung and was a current smoker with a 30-pack-year smoking history with no prior history of thoracic RT. This patient had been previously diagnosed with chronic obstructive pulmonary disease and was on oxygen prior to RT. The patient did not have a clinical diagnosis of ILD at the time of recruitment and enrollment, but it was noted that the patient had radiographic and respiratory evidence suggestive of ILD. After treatment initiation, the patient was classified as having subclinical ILD at the time of his lung cancer diagnosis. SBRT was administered as 50 Gy in 5 fractions given every other day; the treatment plan is shown in Fig. 1A. No adjuvant immunotherapy was given. Multimodality imaging was acquired at 3 visits as shown in Fig. 1B (baseline [pre-SBRT], 3 weeks after RT, and 3 months after RT) using an advanced 3T PET/MRI system (GE 750, GE Healthcare) that allowed both dynamic FDG PET and HP-Xe MRI. This approach enabled visualization of the dynamic response of lung function in the immediate post-RT and recovery after RT phases. Thus, metabolic signatures were used to obtain the net uptake rate K_i_ of FDG, and HP-Xe MRI was used to quantify ventilation using the ventilation defect percentage of xenon gas in the airspaces (VDP),^19^^,^^20^ and gas exchange using the ratio of xenon gas uptake in the capillary-tissue interface (“membrane”) to uptake in the red blood cells (RBC:Membrane)^21^ at all 3 timepoints. Importantly, 4DCT^18^^,^^22^, ^23^, ^24^, ^25^ as well as UTE MRI^26^ were acquired to likewise track and compare structural changes in response to RT, such that structure–function relationships could be assessed. A detailed description of image acquisition and analysis can be found in the supplement (Appendix E1). Pulmonary function tests (PFTs), including the diffusing capacity of the lungs for carbon monoxide and spirometry for the forced expiratory volume in 1 second (FEV1) and forced vital capacity (FVC), were completed pre-SBRT and 3 weeks post-SBRT, and at a clinical follow-up visit 6 months post-SBRT.

## Results

**Figure 2:**
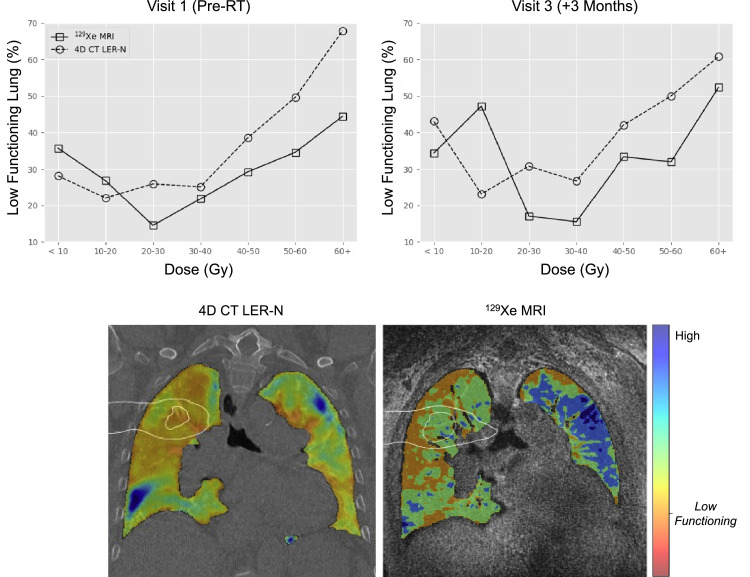
4DCT and HP-Xe MRI-derived ventilation comparison. Ventilation metrics derived by 4DCT LER-N using 10 different respiratory phases and HP-Xe MRI were compared prior to RT and 3 months following RT. Results were reported as the percentage of low-functioning lung volume within each 10-Gy isodose region (top). Ventilation maps 3 months after therapy comparing functional lung derived by 4DCT (bottom left) and HP-Xe MRI (bottom right) are shown, with blue indicating high-functioning lung and orange indicating low-functioning lung; 20-Gy and 10-Gy isodose curves are displayed (white outline overlay). *Abbreviations:* 4DCT = 4-dimensional computed tomography; LER-N = N-phase localized expansion ratio; MRI = magnetic resonance imaging; RT = radiation therapy.

Table 1 summarizes whole-lung measures of VDP, RBC:Membrane, Membrane:Gas, and low-functioning lung defined by 4DCT, K_i_ in the tumor and lung volume, and PFT across all study time points and the 6-month clinical follow-up for relevant measures. As compared to pre-SBRT, Membrane:Gas and K_i_ were decreased at 3 weeks post-SBRT (Membrane:Gas: pre-SBRT = 1.10 ± 0.40, 3 weeks = 0.90 ± 0.30, D = 0.2 ± 0.5; K_i_: pre-SBRT = 1.3 × 10^−3^ min^−1^, 3 weeks = 0.43 × 10^−3^ min^−1^, D = 0.87 × 10^−3^ min^−1^), but not at 3 months post-SBRT (Membrane:Gas: pre-SBRT = 1.10 ± 0.40, 3 months = 1.00 ± 0.50, D = 0.1 ± 0.64; K_i_: pre-SBRT = 1.30 × 10^−3^ min^−1^, 3 months = 0.74 × 10^−3^ min^−1^, D = 0.56 × 10^−3^ min^−1^). VDP was increased at 3 weeks post-RT (pre-SBRT = 26.0%, 3 weeks = 40.1%, D = 14.1%) but returned to near pre-SBRT measures at 3 months post-SBRT (3 weeks = 40.1%, 3 months = 28.3%, D = 11.8%). 4DCT-derived ventilation also showed an increase in low-functioning lung after RT and is regionally consistent with VDP as described below, although with a systematically higher estimate of poorly functioning lung (Fig. 2).

**Table 1:** PFTs, HP-Xe, and ^18^F-FDG PET MRI whole-lung measures

Parameter	Visit 1 *Pre - RT*	Visit 2 *+3 weeks*	Visit 3 *+3 months*	Clinical visit *+6 months*
FVC (%p)	69	93	-	77
FEV1 (%p)	80	93	-	78
DLCO (%p)	18	34	-	17
Low-functioning (%) 4DCT	27.9	-	41.5	-
VDP (%)	26.0	40.1	28.3	-
RBC:Membrane	0.15 ± 0.09	0.15 ± 0.11	0.11 ± 0.11	-
Membrane:Gas (%)	1.10 ± 0.40	0.90 ± 0.30	1.00 ± 0.50	-
K_i_ (min^−1^) $\times \;\;10^{-3}$ *(Lung volume)*	1.30	0.43	0.74	-
K_i_ (min^−1^) $\times \;\;10^{-3}$ *(Tumor volume)*	39.18	20.80	6.62	-

*Abbreviations:* 4DCT = four-dimensional computed tomography; DLCO = diffusing capacity of the lungs for carbon monoxide; FEV1 = forced expiratory volume in 1 second; FVC = forced vital capacity; K_i_ = net influx constant; PET = positron emission tomography; PFT = pulmonary function test; RBC = red blood cell; VDP = ventilation defect percentage.

### Regional dose-dependent analysis

**Figure 3:**
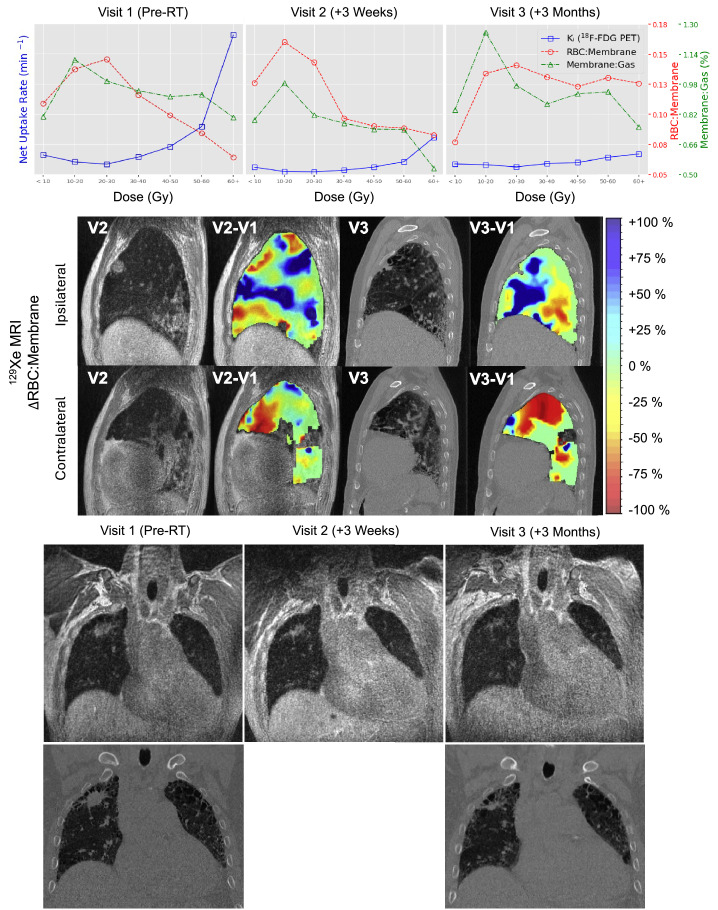
^18^F-FDG PET, HP-Xe MRI longitudinal and structural changes. ^18^F-FDG PET and HP-Xe MRI-derived metrics were compared (first row) before RT (left), and 3 weeks (middle) and 3 months after RT (right). Net influx constant, K_i_, describing the amount of irreversible ^18^F-FDG uptake is shown in blue, RBC:Membrane in red, and Membrane:Gas in green. Spatial maps depicting changes in RBC:Membrane with respect to the baseline visit (second row) are displayed with increases in RBC:Membrane shown in blue and decreases in red. UTE MR images (row 4) before RT (left), 3 weeks after RT (middle), and 3 months after RT (right) are shown. CT images (row 5; bottom) are shown for pre-RT (left) and 3 months post-RT (right). Changes in the tumor volume are depicted in both UTE and CT. Fibrotic changes near the tumor are observed 3 months post-RT. *Abbreviations:* 4D = 4-dimensional; CT = computed tomography; ^18^F-FDG = fluorodeoxyglucose; MRI = magnetic resonance imaging; PET = positron emission tomography; RT = radiation therapy; UTE = ultra short time to echo.

**Figure 4:**
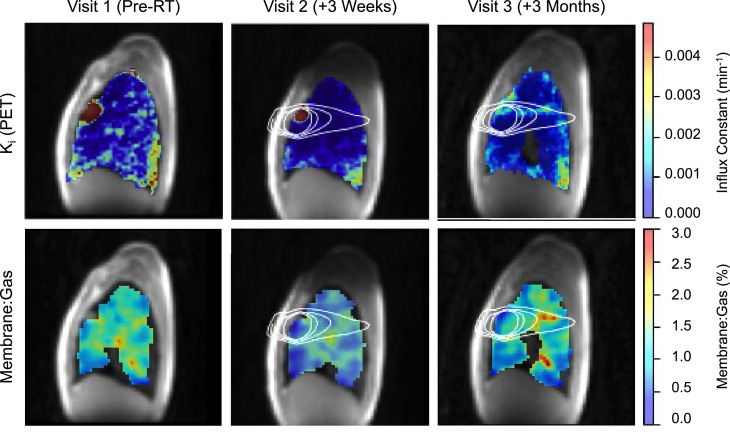
Net influx constant (K_i_) and membrane:gas spatial maps. Spatial maps describing ^18^F-FDG (top) and HP-Xe (bottom) uptake in the lung tissues overlayed on ^1^H MRI before, and 3 weeks and 3 months following, completion of RT. The 10-, 20-, 30-, and 40-Gy isodose curves are shown. Decreased ^18^F-FDG and Membrane:Gas is observed across the lung 3 weeks after therapy (middle column). Increased ^18^F-FDG and Membrane:Gas is observed in the beam path between 10 and 20 Gy isodose curves 3 months after RT (right column). *Abbreviations:*^18^F-FDG = fluorodeoxyglucose; PET = positron emission tomography; RT = radiation therapy.

HP-Xe MRI, 4DCT, and associated uncertainties of RBC:Membrane, Membrane:Gas, and K_i_ measurements within each isodose volume at each visit are summarized in Table 2. Figure 2 compares regional lung ventilation derived by 4DCT and HP-Xe MRI pre-SBRT and 3 months post-SBRT, showing low-functioning lung predominantly in the high-dose region with similarities in regional variation across the dose distribution. In addition, Fig. 2 displays 4DCT and HP-Xe MRI spatial maps qualitatively comparing the change in measurements 3 months post-SBRT, with the 10 to 20-Gy isodose curves overlayed in white; note the 1 region of discordance in low-functioning lung was for 10 to 20-Gy region at 3 months after RT where VDP was substantially greater than 4DCT low-functioning lung. This region corresponded to an area of elevated RBC:Membrane and Membrane:Gas pre-SBRT that may be compensatory in response to the tumor in the apical lung and other comorbidities (ie, emphysema and fibrotic lung disease) visible most prominently in the lung bases and contralateral lung. After RT, HP-Xe MRI measures suggested a redistribution of ventilation, and RBC:Membrane across the 10-Gy bins with increased gas exchange observed especially in high-dose (>30 Gy) regions, and decreased gas exchange in regions that received < 10 Gy (Fig. 3). Aside from the reduced uptake because of treatment response observed in the tumor region, K_i_ showed similar patterns to the Membrane:Gas across the lungs including the overall reduction 3 weeks after RT and increase within the beam path 3 months after RT (Fig. 4). Membrane:Gas in the beam path at 3 months, primarily within the 10 to 20-Gy isocontours (Fig. 4), was substantially elevated compared to Membrane:Gas in the volume of lung receiving < 10 Gy (Table 2).

**Table 2:** Regional 4DCT, HP-Xe MRI, and ^18^F-FDG PET MRI-derived metrics

Dose (Gy)	< 10			10-20			20-30			30-40			40-50			50-60			60+		
Visit	1	2	3	1	2	3	1	2	3	1	2	3	1	2	3	1	2	3	1	2	3
Lung volume (mL)	3207.8	-	2821.5	216.8	-	205.7	78.6	-	74.7	33.0	-	31.0	17.1	-	15.7	14.9	-	13.4	10.9	-	29.6
Low-functioning (%) 4DCT	28	-	43	22	-	23	25	-	30	38	-	26	49	-	42	67	-	50	27	-	60
VDP (%)	35	46	34	26	44	47	14	18	17	21	14	15	29	17	33	34	18	31	44	20	52
RBC: Membrane	0.11 ± 0.02	0.12 ± 0.03	0.07 ± 0.02	0.14 ± 0.03	0.16 ± 0.04	0.13 ± 0.02	0.14 ± 0.02	0.14 ± 0.02	0.14 ± 0.02	0.11 ± 0.09	0.09 ± 0.01	0.13 ± 0.02	0.10 ± 0.01	0.09 ± 0.01	0.12 ± 0.01	0.08 ± 0.01	0.09 ± 0.01	0.13 ± 0.02	0.06 ± 0.01	0.08 ± 0.01	0.12 ± 0.02
Membrane: Gas (%)	0.80 ± 0.19	0.79 ± 0.13	0.84 ± 0.20	1.11 ± 0.15	0.98 ± 0.15	1.25 ± 0.18	0.99 ± 0.11	0.81 ± 0.08	0.97 ± 0.10	0.94 ± 0.07	0.77 ± 0.07	0.87 ± 0.09	0.91 ± 0.07	0.74 ± 0.05	0.92 ± 0.08	0.92 ± 0.08	0.73 ± 0.06	0.94 ± 0.11	0.80 ± 0.12	0.53 ± 0.05	0.75 ± 0.13
K_i_ (min^−1^) $\times \;\;10^{-3}$	1.29 ± 0.27	0.49 ± 0.17	0.70 ± 0.29	0.82 ± 0.02	0.19 ± 0.02	0.65 ± 0.02	0.68 ± 0.06	0.16 ± 0.03	0.51 ± 0.04	1.15 ± 0.14	0.28 ± 0.11	0.72 ± 0.12	1.84 ± 0.31	0.49 ± 0.21	0.81 ± 0.22	3.17 ± 0.49	0.85 ± 0.27	1.14 ± 0.28	9.25 ± 1.95	2.47 ± 0.85	1.36 ± 0.36

*Abbreviations:* 4DCT = 4-dimensional computed tomography; K_i_ = net influx constant; LER-N = N-phase local expansion ratio; MRI = magnetic resonance imaging; PET = positron emission tomography; RBC = red blood cells; VDP = ventilation defect percentage.

Low-functioning lung derived from 4DCT LER-N is reported as the percentage of volume within each isodose volume (0-10, 10-20, etc) having a local expansion ratio < 1.2. HP-Xe MRI ventilation is reported as the percentage of volume within each isodose volume classified as VDP. RBC:Membrane, Membrane:Gas, and K_i_ are reported as (mean ± SD) for each isodose volume.

### Structural changes and patient outcome

The following structural observations were retrospectively made by an experienced cardio-thoracic radiologist, after the conclusion of all study visits and clinical follow-up at 6 months, using UTE MRI and confirmed using CT. Prior to RT, moderate-to-severe emphysema was observed predominantly in the upper lobes of the lung, with severe interstitial lung abnormalities (ILAs) in the lower lobes with honey combing, and small ground glass opacity directly adjacent to the tumor. Three weeks after RT, the lesion decreased in size, but was accompanied by increases in ground glass opacity directly adjacent to the tumor. Three months after RT, the tumor substantially decreased in size, flattening, with increased fibrotic changes surrounding the tumor.

The patient was not clinically diagnosed with RP but passed away approximately 2 years (779 days) after the conclusion of external beam treatment. The cause of death was listed as ILD.

## Discussion

This is the first multimodality comparison of functional lung imaging methods made possible by PET/MRI technology and advanced chest CT analysis. HP-Xe MRI measures of pulmonary function were compared with FDG PET uptake rate and 4DCT measures in a lung cancer patient, prior to and following standard RT treatment. Importantly, posttreatment alterations observed using HP-Xe MRI were in qualitative agreement with more established techniques for evaluating lung inflammation (FDG PET) and ventilation (4DCT). Moreover, there were substantial dynamics of functional change at 3 weeks post-RT that suggest that functional changes in the lungs in response to RT should be considered in more detail. Additionally, this investigation suggests that the ventilation and gas exchange information provided by HP-Xe and UTE MRI could be a practical and informative adjunct for monitoring of RT treatment, without the additional ionizing radiation required for PET or CT techniques.

This individual case study demonstrates changes in regional lung function with RT in a lung cancer patient with significant comorbidities, inclusive of areas of emphysema and extensive interstitial fibrosis which were present prior to therapy initiation. Monitoring regional patterns of lung function in the context of such structural disease patterns is likely helpful in understanding both the response of pathophysiologic mechanisms, and possible predictors, of lung injury because of RT as well as outcomes of pneumonitis.^23^ Although we do not yet understand the complex interplay between pre-existing ILAs with RT, previous investigations by our group provides important context about gas exchange in and near fibrotic lung regions. HP-Xe MRI of gas exchange is sensitive to early changes in ILD progression and treatment using anti-fibrotic therapy.^13^^,^^27^, ^28^, ^29^ More specifically, gas exchange ratios are reduced in ILD patients compared to matched healthy controls, particularly in regions of the lungs with overt fibrotic injury on CT. This evidence supports the notion that this patient likely had regions of compromised pulmonary function prior to RT treatment, which likely impacted the physiological response. Further study of the regional functional response to RT is warranted because of the complexity of the studied individuals’ response to RT. Furthermore, the redistribution of functional regions after treatment suggests modifying the treatment plan based on patient-specific patterns of regional physiology may be beneficial.

The eventual complications of fibrotic injury in the tumor region/RT beam path revealed through both HP-Xe and UTE MRI, alongside the progression of ILD resulting in the death of the patient, aligns with elevated risk for RILI and fibrosis in patients with pre-existing ILD.^30^^,^^31^ It is important to note that patients with pre-existing ILD are often referred to RT treatment because of the associated complications of surgical intervention.^32^ Surprisingly, this patient was not formally diagnosed with RILI. These imaging findings support the notion that integrating imaging into RP diagnosis and monitoring may improve the reliability of RILI severity grading systems and help identify patients who may benefit from earlier and more aggressive intervention.

Feasibility studies using HP-Xe MRI to guide treatment planning and monitoring of RT in lung cancer patients are ongoing.^12^^,^^33^ Assessment of regional lung ventilation and gas exchange pre-RT offers the opportunity to use functional avoidance to spare healthy tissue, and depending on location of the tumor, increase dose to the tumor region. In concert with pre-RT planning, active monitoring offers the potential to update the treatment plan during treatment and improve understanding of the pathophysiological mechanisms and measures associated with RP and outcomes of chronic lung injury.

## Conclusions

HP-Xe MRI was successfully acquired in a lung cancer patient undergoing RT longitudinally in conjunction with more established measures of regional lung function. The technique was well tolerated and revealed patterns of redistribution of measures related to inflammation and gas exchange before and after therapy. HP-Xe MRI may be a useful tool for longitudinal monitoring and guidance of RT in lung cancer.

## Disclosures

Sean B. Fain reports financial support was provided by National Institutes of Health. Sean B. Fain reports financial support was provided by University of Wisconsin-Madison Carbone Cancer Center. Sean B. Fain reports a relationship with GE Healthcare that includes: funding grants. Sean B. Fain reports a relationship with Polarean LLC that includes: consulting or advisory, funding grants, and speaking and lecture fees. Sean B. Fain has patent #US-10677874-B2 issued to Wisconsin Alumni Research Foundation. Scott B. Perlman is a consultant for GE Healthcare and serves on the Scientific Advisory Board of AIQ Solutions. Andrew D. Hahn is a consultant for Polarean LLC and receives research support from GE Healthcare. If there are other authors, they declare that they have no known competing financial interests or personal relationships that could have appeared to influence the work reported in this paper

## Appendix Supplementary materials

Supplementary_information_annonymized Image, application 1

## Footnotes

Supplementary material associated with this article can be found in the online version at doi:10.1016/j.adro.2025.101810.
