# Supplementary material for: Functional Imaging of Changes in Lung Function Before and After Radiation Therapy of Lung Cancer
Source: Adv Radiat Oncol. 2025 Jun 20;10(8):101810. doi: 10.1016/j.adro.2025.101810 (PMC12221846; doi:10.1016/j.adro.2025.101810)
Supplement: Supplementary_information_annonymized [file mmc1.docx]

**Appendix E1**

**Methods**

*^129^Xe and PET/MRI Acquisition*

Isotopically enriched (85%) ^129^Xe was polarized using a Polarean 9820 rubidium spin-exchange optical pumping system. Three ^129^Xe doses were prepared for each visit, including calibration, ventilation, and gas exchange doses. All three doses were supplemented with N_2_ to achieve a volume of 20% of the subject’s forced vital capacity (FVC). The subject was positioned feet first supine with their arms by their side and fitted with a flexible ^129^Xe transmit/receive chest coil in the 3T GE 750 multinuclear-capable PET/MRI. Under breath-hold, the calibration dose of ^129^Xe acquired whole-lung spectroscopy measuring dissolved and gas-phase spectra at repetition time (TR) equal to 15 ms and with a flip angle of 20°.^1, 2^ From the calibration dose, the ^129^Xe resonance frequency, transmit voltage, and time to echo (TE) corresponding to the condition where the red blood cells (RBC) and membrane signals are 90° out of phase (TE90) were determined using an automatic fitting routine built into a prototype multi-nuclear spectroscopy package (FIDALL, GE Healthcare). Ventilation images were acquired using 2D slice selective imaging with Cartesian encoding accomplished with a fast gradient echo sequence. Using this technique, 14-18 slices were collected at each visit with an in-plane resolution of 1.875 mm by 1.875 mm providing maps of the ventilation distribution throughout the lungs. Gas exchange images were obtained using a radial 3D sequence to acquire gas and dissolved phase ^129^Xe signals. Both phases were sampled with 1000 radial projections each in an interleaved fashion^1, 2^. Gas and dissolved phase flip angles were 0.5^°^ and 20°, respectively, with the subject-specific TE90 found during the previously acquired calibration scan.^1, 2^ An anatomic ^1^H MRI was acquired during a breath-hold of room air with the same inhalation volume used in the ^129^Xe MRI. Finally, a free breathing ^1^H three-dimensional radial UTE MRI with 1.25 mm isotropic resolution was completed.^3^ Dynamic ^18^F-FDG PET data was continuously acquired for 60-minutes following a single bolus of 10.54 mCi at Visit 1, 9.89 mCi at Visit 2, and 7.72 mCi at Visit 3.

*^129^Xe and PET/MRI Reconstruction and Analysis*

The anatomic ^1^H MR images and ^129^Xe ventilation images were corrected for bias field (B1) heterogeneity using the N4BiasFieldCorrection within the Advanced Normalization Tools. Voxels were then clustered into ventilation defect percentage (VDP), low-, medium-, or high-ventilation (LVP, MVP, HVP) using adaptive *K*-means to assess ventilation of airspaces based on the signal intensity exhibited throughout the thoracic cavity.^4, 5^ Using the 1-point Dixon method,^1, 2^ the dissolved xenon signal was split into its two components, specifically xenon in the interstitial tissues of the alveolar wall (Membrane) and xenon dissolved in the RBC. The Membrane and RBC signals are then normalized to the gas signal on a voxel-by-voxel basis, yielding spatial maps of Membrane:Gas and RBC:Gas uptake, and the RBC:Membrane ratio, a measure of gas exchange.

Reconstruction parameters for dynamic PET were as follows: 60-second frame duration, 45 cm FOV, matrix 256 x 256 x 89, voxel spacing 2.34 x 2.34 x 2.78, 2 iterations, VPFX-S, time-of-flight, and 5 mm smoothing. PET images were manually segmented using ITK-Snap software. The time-activity curves of the tumor and lung ROIs were normalized by the time-activity curve of the blood pool located in the left ventricle, to assess relative FDG uptake in the three-dimensional ROIs in the tumor and lung, yielding a nearly linear time-activity ratio function. Patlak analysis was used to determine the net uptake rate *K_i_* in units of min^-1^ of 18F-FDG in the tumor region and lung parenchyma using MATLAB (Mathworks, Natick, MA).

An experienced cardio-thoracic radiologist (RMB) evaluated ^1^H UTE MR images for tumor and lung ROIs, fibrotic injuries, and structural changes pre- and post-SBRT.

*4DCT Acquisition, Post-Processing, and Ventilation Derivation*

Using the Siemens SOMATOM Definition Edge (Siemens Healthineers) 4DCT, the subject was imaged while positioned head-first supine with their arms positioned above their head using scan parameters of 120 kV, 100 mAs/rotation, 0.09 pitch, 0.5 second tube rotation time, 76.8 mm beam collimation, and 128 detector rows. Using the Varian Real-Time Position Management (RPM) system (Varian Medical Systems Inc.), respiratory gating was performed. From this, 10 breathing phase volumes, five inspiratory (IN) and five expiratory (EX), were reconstructed in 20% increments where 100IN represents end inspiration and 0EX end expiration using a medium smooth kernel (B50f), 512 mm extended field of view and 1 mm slice thickness.^6, 7^ Ventilation maps representing regional variation in lung function were generated using the N-phase local expansion ratio (LER-N) technique as described.^6^ Briefly, breathing phase images were registered to the 0EX image using B-spline deformable image registration with a sum of squared tissue volume differences metric.^8^ Next, the Jacobian (expansion/contraction of each voxel relative to 0EX) was calculated for all nine (N-1) breathing phases to determine the LER-N ratio.^9^ Voxels in the lung having out-of-phase ventilation, reaching minimum or maximum expansion in a different phase than 100IN, are accounted for.^6^

*Treatment Planning*

Using the average 4DCT image, an experienced thoracic radiation oncologist (AMB) contoured the internal (ITV) and clinical (CTV) target volume and organs at risk (OAR) using MIM (MIM Software Inc) which were subsequently utilized in treatment planning using Raystation (RaySearch Laboratories) with the planning target volume (PTV) created by a 5 mm expansion on the CTV.^10^ Target goals were a minimum 98% coverage of the prescription dose to the PTV, a mean PTV dose greater than or equal to 120% of the prescription dose, and a minimum of 90% of the ITV being covered by 120% of the prescription dose.^10^ OAR dose constraints include those to the heart, esophagus, spinal cord, and lungs. Additional details on the radiation treatment planning are previously described.^10^

*Image Registration*

The subject’s ^1^H and ^129^Xe MRIs were inherently aligned due to the acquisition of both taking place in the same session. Rigid registration was used to correct patient motion between scans. ^1^H, corresponding ^129^Xe MRI ventilation maps and dissolved signals were then registered to the ^1^H UTE MRI using an affine registration. From there, the ^1^H, ^129^Xe, and ^1^H UTE MRIs for each visit were registered to the subject’s RT planning 4DCT scans using an affine registration. Following this, K_i_ maps from the ^18^F-FDG PET MRI were registered to the ^1^H UTE MRI using a mask-to-mask affine registration permitting the evaluation of *K*_i_ and ^129^Xe MRI measures regionally co-localized to the treatment plan radiation dose distribution map.

*Data Analysis*

^129^Xe ventilation, Membrane:Gas, and RBC:Membrane means and standard deviations were assessed in the lungs both holistically and regionally by dose delivered in 10 Gy increments. The net uptake of ^18^F-FDG PET was evaluated in the tumor and the lung ROIs each segmented in ITK-Snap. 4D CT-derived ventilation maps were computed and compared to ^129^Xe ventilation maps at Visits 1, before RT, and 3 following RT. HVP, MVP, LVP, and VDP were normalized in volumes of lung defined in 10 Gy increments. Ventilation metrics were reported as “Low Functioning Lung” describing the percentage of lung volume within each of the 10 Gy isodose volumes classified as VDP in ^129^Xe maps or having a local expansion ratio of less than 1.2; this local expansion ratio was empirically derived from the cohort population average found to be most predictive of RP.^6^ Means and standard deviations of Membrane:Gas, RBC:Membrane, and *K_i_* were estimated for each 10 Gy isodose volume using Bayesian conditional autoregressive regression (CAR) models to account for spatial correlations. Models were fit with the R-INLA software (Bivand, Gómez-Rubio, and Rue 2015). Statistical analysis was conducted with version 4.4.0 of the R software (Vienna, Austria). Only voxels within the lung ROI were considered (^1^H UTE MR-contoured lung with the exclusion of ^1^H UTE MR-contoured tumor) in the analysis.

**References**

**1.** Kaushik SS, Robertson SH, Freeman MS, et al. Single-breath clinical imaging of hyperpolarized (129)Xe in the airspaces, barrier, and red blood cells using an interleaved 3D radial 1-point Dixon acquisition. *Magn Reson Med.* 2016;75:1434-1443.

**2.** Niedbalski PJ, Hall CS, Castro M, et al. Protocols for multi-site trials using hyperpolarized. *Magn Reson Med.* 2021;86:2966-2986.

**3.** Johnson KM, Fain SB, Schiebler ML, Nagle S. Optimized 3D ultrashort echo time pulmonary MRI. *Magn Reson Med.* 2013;70:1241-1250.

**4.** He M, Zha W, Tan F, Rankine L, Fain S, Driehuys B. A Comparison of Two Hyperpolarized. *Acad Radiol.* 2019;26:949-959.

**5.** Mummy DG, Kruger SJ, Zha W, et al. Ventilation defect percent in helium-3 magnetic resonance imaging as a biomarker of severe outcomes in asthma. *J Allergy Clin Immunol.* 2018;141:1140-1141.e1144.

**6.** Flakus MJ, Kent SP, Wallat EM, et al. Metrics of dose to highly ventilated lung are predictive of radiation-induced pneumonitis in lung cancer patients. *Radiother Oncol.* 2023;182:109553.

**7.** Han D, Bayouth J, Bhatia S, Sonka M, Wu X. Characterization and identification of spatial artifacts during 4D-CT imaging. *Med Phys.* 2011;38:2074-2087.

**8.** Cao K, Ding K, Reinhardt JM, Christensen GE. Improving Intensity-Based Lung CT Registration Accuracy Utilizing Vascular Information. *Int J Biomed Imaging.* 2012;2012:285136.

**9.** Shao W, Patton TJ, Gerard SE, et al. N-Phase Local Expansion Ratio for Characterizing Out-of-Phase Lung Ventilation. *IEEE Trans Med Imaging.* 2020;39:2025-2034.

**10.** Baschnagel AM, Flakus MJ, Wallat EM, et al. A Phase 2 Randomized Clinical Trial Evaluating 4-Dimensional Computed Tomography Ventilation-Based Functional Lung Avoidance Radiation Therapy for Non-Small Cell Lung Cancer. *Int J Radiat Oncol Biol Phys.* 2024.
